# Blood-derived miRNA levels are not correlated with metabolic or anthropometric parameters in obese pre-diabetic subjects but with systemic inflammation

**DOI:** 10.1371/journal.pone.0263479

**Published:** 2022-02-04

**Authors:** Prabu Paramasivam, Emmanuelle Meugnier, Kuppan Gokulakrishnan, Harish Ranjini, Lisa R. Staimez, Mary Beth Weber, K. M. Venkat Narayan, Hubert Vidal, Nikhil Tandon, Dorairaj Prabhakaran, Anjana Ranjit Mohan, Viswanathan Mohan, Sophie Rome, Muthuswamy Balasubramanyam

**Affiliations:** 1 Madras Diabetes Research Foundation and Dr. Mohan’s Diabetes Specialities Centre, WHO Collaborating Centre for Non-Communicable Diseases Prevention and Control & IDF Centre of Education, Gopalapuram, Chennai, India; 2 Department of Neurology, School of Medicine, University of New Mexico Health Sciences Center, Albuquerque, NM, United States of America; 3 CarMeN Laboratory, French National Institute of Health and Medical Research (INSERM) U1060, National Research Institute for Agriculture, Food and Environment (INRAE) U1397, University of Lyon, Claude Bernard University Lyon 1, Oullins, France; 4 Department of Neurochemistry, National Institute of Mental Health and Neurosciences (NIMHANS), Bengaluru, India; 5 Emory University, Atlanta, GA, United States of America; 6 Department of Endocrinology, All India Institute of Medical Sciences, New Delhi, India; 7 Centre for Chronic Disease Control and Public Health Foundation of India, New Delhi, India; 8 Medical & Health Sciences (MHS), SRM Institute of Science and Technology (SRMIST), Chennai, India; Università degli Studi di Milano, ITALY

## Abstract

As blood-derived miRNAs (c-miRNAs) are modulated by exercise and nutrition, we postulated that they might be used to monitor the effects of a lifestyle intervention (LI) to prevent diabetes development. To challenge this hypothesis, obese Asian Indian pre-diabetic patients were submitted to diet modifications and physical activity for 4 months (LI group) and compared to a control group which was given recommendations only. We have considered 2 periods of time to analyze the data, *i*.*e*.; a first one to study the response to the intervention (4 months), and a second one post-intervention (8 months). At basal, 4 months and 8 months post-intervention the levels of 17 c-miRNAs were quantified, selected either for their relevance to the pathology or because they are known to be modulated by physical activity or diet. Their variations were correlated with variations of 25 metabolic and anthropometric parameters and cytokines. As expected, fasting-glycaemia, insulin-sensitivity, levels of exercise- and obesity-induced cytokines were ameliorated after 4 months. In addition, the levels of 4 miRNAs (*i*.*e*.; miR-128-3p, miR-374a-5p, miR-221-3p, and miR-133a-3p) were changed only in the LI group and were correlated with metabolic improvement (insulin sensitivity, cytokine levels, waist circumference and systolic blood pressure). However, 8 months post-intervention almost all ameliorated metabolic parameters declined indicating that the volunteers did not continue the protocol on their own. Surprisingly, the LI positive effects on c-miRNA levels were still detected, and were even more pronounced 8 months post-intervention. In parallel, MCP-1, involved in tissue infiltration by immune cells, and Il-6, adiponectin and irisin, which have anti-inflammatory effects, continued to be significantly and positively modified, 8 months post-intervention. These data demonstrated for the first time, that c-miRNA correlations with metabolic parameters and insulin sensitivity are in fact only indirect and likely associated with the level systemic inflammation. More generally speaking, this important result explains the high variability between the previous studies designed to identify specific c-miRNAs associated with the severity of insulin-resistance. The results of all these studies should take into account the level of inflammation of the patients. In addition, this finding could also explain why, whatever the pathology considered (*i*.*e*.; cancers, diabetes, neurodegenerative disorders, inflammatory diseases) the same subset of miRNAs is always found altered in the blood of patients *vs* healthy subjects, as these pathologies are all associated with the development of inflammation.

## Introduction

MiRNAs are a class of evolutionally conserved small noncoding RNAs which function as negative regulators of gene expression [1]. Since 2008 [2], several independent groups have described the presence of significant amounts of circulating miRNAs (c-miRNAs) in extracellular human body fluids where they are remarkably stable. A significant number of studies have demonstrated that the levels of blood-derived c-miRNAs are modified in diabetic patients [3, 4] and more importantly, that alterations of c-miRNA levels already occurred in pre-diabetic state patients with Caucasian or Indian/Asian phenotypes [3, 5], suggesting that they could be used as early biomarkers of type 2 diabetes (T2DM) [5, 6]. Interestingly, it was shown that c-miRNA levels were modulated during treatments/surgery to restore insulin-sensitivity [7–11]. In addition, c-miRNAs are dynamically regulated by exercise training [12], nutrition supplementations [13], or diet restriction [14, 15] in diabetic subjects. However, when we analysed carefully all data from literature from the last 15 years, it was clear that until now, no consensual c-miRNA signature has been identified as a good biomarker to follow the development of diabetes, in pre-diabetic patients, or to classify the patients according to the severity of the disease [3]. More importantly, all c-miRNAs so far found to be modified in the blood of diabetic patients vs healthy subjects, or modulated by lifestyle interventions, are also found modulated in the blood of patients suffering from other diseases such as cancers, neurodegenerative diseases or inflammation, which indicates that their variations are common to various pathologies and not restricted to the diabetes condition. Finally, we also found conflicting results showing the up-regulation of some c-miRNAs in patients with diabetes vs healthy subjects, that were down regulated in other studies or not affected at all [3]. Although we cannot exclude some technical aspects associated to the extraction and quantification of c-miRNAs which could explain these discrepancies, or that the groups of patients selected for these studies are sometime very different between publications (BMI, sex, age, ethnicity), these observations forced us to re-evaluate the use of c-miRNAs as blood biomarkers, and to explain the origin of their blood variations. Therefore, in this study, we have conducted a second study within an existing interventional study, consisting in two groups of obese pre-diabetic Indian patients that were either submitted to a global lifestyle intervention protocol (LI) (i.e.; diet modification combined with physical activity) for 4 months, or left without any intervention during the same period of time [16]. After 4 months, all subjects were left to their own devices with minimal contacts with clinicians for an additional 8 months. During all the protocol (basal, 4 and 8 months post-intervention) the levels of 16 c-miRNAs were quantified, selected either for their relevance for the pathology [3, 5, 7] or because they are known to be modulated by physical activity [17–19] or diet [15]. C-miRNAs variations were correlated with the variations of metabolic/anthropometric parameters and cytokines, at different time points of the protocol. For the first time, the dynamic regulation of serum c-miRNAs was followed during an intervention protocol, but also after a long-time period post-intervention, for the same individuals.

## Materials and methods

### Patient characteristic

Data and samples from sixty Asian Indian obese pre-diabetic patients were selected from one of the intervention studies of the Madras Diabetes Research Foundation (Chennai, India). They belong to the D-CLIP study (Diabetes Community Lifestyle Improvement Program, Clinicaltrial.gov NCT01283308) [16, 20] designed to test the effectiveness of stepwise diabetes program [16]. None of them were on medications at the start of the program. At baseline state, questionnaires permitted to measure habits, quality of life and food frequency of the participants. Anthropometric measurements including height, weight and waist circumstance were obtained using standardized techniques [5]. For this study, fasting serum samples from 5ml of blood were obtained at basal state, 4 months and one year by standard venepuncture using Vacutainer Plus Plastic Serum and SST Tubes (Becton-Dickinson, Franklin, lakes, NJ). Metabolic parameters were measured as previously detailed [5]. IL-6, adiponectin, MCP, TNF-alpha, Irisin and BDNF were quantified with ELISA tests [20]. At baseline, metabolic parameters did not differ significantly between the 2 groups of pre-diabetic individuals, except for the age (S1–S3 Tables).

### Active Lifestyle Intervention (LI) program

The LI group (n = 30) attended 4 months of diet, exercise and weight loss education, and afterwards received metformin (500mg, twice daily) only if they remained at a markedly high risk of conversion from pre-diabetic to diabetic state (individuals with both IFG and IGT or IGF+HbA1C >5.7 n = 8 in this study). During the 4-month active intervention, the LI participants were taught ways to increase physical activity (>150min weekly of moderate-intensity exercise)/decreased sedentary activities, with the goal of >7% weight loss. Participants were trained on improving diet quality (increase intake of high-fiber food and reduction of dietary carbohydrate and fat consumption) and on reducing dietary intake through keeping weekly food diaries. After 4 months active intervention phase, there was a 2 months maintenance phase wherein the participants had lesser contact with the research team but still attended classes, that were less frequent. Pre-diabetic individuals from the control arm, i.e.; standard care (SC) group (n = 30), had a single day with one-on-one visit with a physician, a dietician and a fitness trainer and underwent one group class on diabetes prevention (i.e.; diet quality and physical activity). Metformin prescription for diabetes prevention is not standard of care at the study site, so no control subjects received metformin. Aside from follow-up collection visits, control participants had no additional contact with clinical staff. For this study, we used samples collected at baseline and 4 months as well as 8 months post-intervention.

### C-miRNA isolation

Total RNAs from frozen sera collected at baseline, 4 and 8 months were isolated using miRneasy mini kit (Qiagen, Valencia, CA). Briefly, 750 μl of QIAzol master mix along with 1.25 ul of 0.8ug/μl MS2 carrier RNA (Roche), 3.5 μl of 1.6 x 108 copies/μl of a spike synthetic cel-miR-9 (Qiagen, Valencia, CA) were added to 200 μl of serum samples, vortexed for 30s and incubated for 10 min at room temperature for complete denaturation. Then 200 μl of chloroform was added to each sample, vortexed and incubated for 10 min for complete dissociation of nucleoprotein, followed by spin down at 12,000g for 15 min at 4°C. A fixed volume of the upper aqueous phase was then transferred to 1.5 volume of 100% ethanol to precipitate total RNA. Total RNA was eluted from miRneasy column using 50μl of nuclease free water and stored at -80°c. First strand cDNA synthesis and miRNA quantification by qPCR: cDNA was synthesized from 10 ng of total RNA using the Universal miRCURY LNA microRNA PCR, Polyadenylation and cDNA synthesis kit II. The levels of 17 c-miRNAs were quantified (hsa-miR-146a-3p, hsa-miR-222-3p, hsa-miR-126-3p, hsa-miR-128-3p, hsa-miR-133a-3p, hsa-miR-210-3p, hsa-miR-1-3p, hsa-miR-133b, hsa-miR-423-5p, hsa-let-7d-3p, hsa-miR-21-5p, hsa-miR-29b-5p, hsa-miR-29c-3p, hsa-miR-374a-5p, hsa-miR-130b-3p, hsa-miR-192 and hsa-miR-193). Hsa-miR-222-3p, hsa-miR-126-3p, hsa-miR-210-3p, hsa-let-7d-3p, hsa-miR-210-3p were altered in blood of diabetic subjects and reversely modulated by physical activity or insulin sensitizer [3, 4, 15]. Hsa-miR-423-5p, hsa-miR-192 and hsa-miR-193 were affected in obese patients or T2DM patients and reversely modulated by weight loss [3, 4, 15]. Hsa-miR-133a-3p, hsa-miR-1-3p and hsa-miR-133b were modulated by diet [15]. Hsa-miR-128-3, hsa-miR-130b-3p and hsa-miR-374a-5 were affected in the blood of Indian pre- or T2DM subjects vs heathy patients [5], or in Asian T2DM patients for hsa-miR-146a-3p [3]. Hsa-miR-29b-5p and hsa-miR-29c-3p were increased in blood of T2DM patients vs healthy controls [3]. The blood level of all miRNAs was determines by the using miRCURY LNA microRNA PCR system, Exilent SYBR green master mix, using a real-time thermocycler (Light cycler 96, Roche). As miR-192 and miR-193 were not detected in all patients, they were not further considered in the study. miRNA levels were normalized to cel-miR-39-3p. In this paper, we are considering Ct values, which are inversely correlated with miRNA concentrations.

### Statistical analyses

Data were analysed using R (V4.04), lme4 (V1.1–27.1), lsmeans (v 2.30–0) and emmeans (V1.7.0) libraries and are presented as Mean ± standard deviation. p<0.05 were considered as significant values. First normality assessment of the data was performed using Kolmogorov-Smirnov test. If the test was not significant, data were transformed (log2 or sqrt transformation). Comparison of clinical parameters at basal state between SC and LI group was performed using Fisher test for gender, and Student t-test for all other variables. Benjamini Hochberg correction for multiple test was applied. Linear models, taking into account repeated measurement and age and gender as covariates, were used to investigate the effect of time, and interaction of time and treated group. Tukey hsd post hoc test was then applied to identify which group and time point were different. At 4-month lifestyle intervention and 8-month follow-up all data were normalized to the basal state in order to calculate Spearman correlations between variations of miRNA concentrations and variations of metabolic parameters. In order to determine the impact of the treatment with metformin on the results, we also performed statistical analyses removing the data of the 8 treated subjects. When the results with and without these 8 patients were different, the significant values were not considered and not discussed (see Table legends). Very important, these 8 individuals were not significantly different from the other subjects not treated with metformin, when all data were considered at T = 0, T = 4 months and T = 12 months, as shown on S1 Fig.

## Results

### The lifestyle intervention (LI) modifies the metabolic risk factors of pre-diabetic subjects

**Fig 1:**
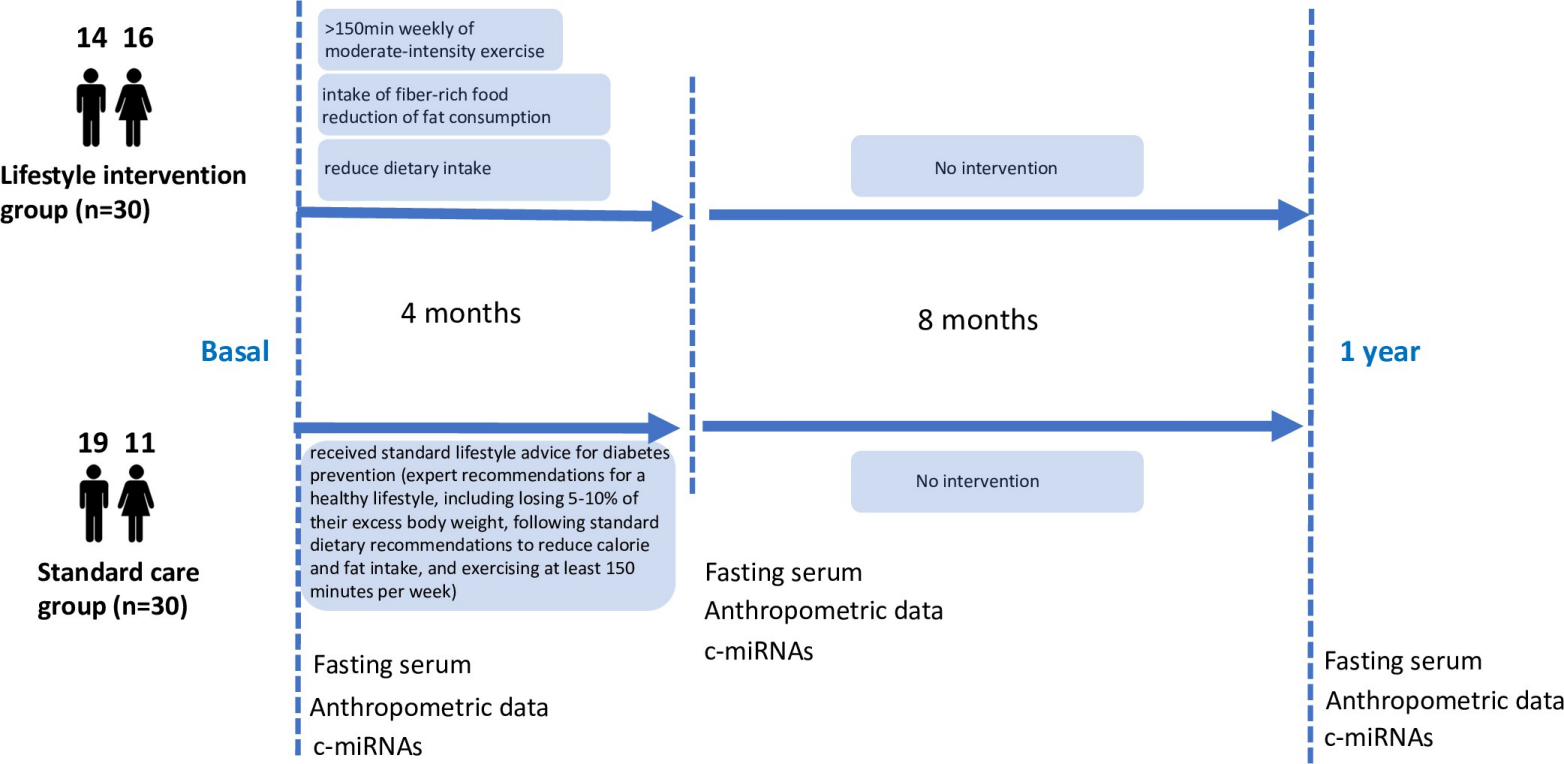
Summary of the protocol showing when metabolic/anthropometric data and c-miRNA/cytokines levels were quantified in serum from obese pre-diabetic Indian patients. All the patients belong to the cohort D-CLIP [16].

We deliberately did not consider normoglycemic subjects in this study as the purpose here was to determine whether c-miRNAs were modulated by LI in obese pre-diabetic patients, and not to identify differently expressed c-miRNAs between controls and diabetic patients. The protocol is summarized in Fig 1. As indicated in S1 Table, all enrolled subjects were over-weighted and had metabolic alterations characteristic of a pre-diabetic state, including hyperinsulinemia, impaired glucose tolerance and were insulin-resistant. Metabolic parameters were then re-analysed after 4 months of intervention (Table 1). As expected the LI group experiences maximal metabolic benefits during the 4-months intervention (Table 1). In contrast, the participants from the standard care (SC) group had modestly decreased their waist-circumference and BMI without consequences on the other metabolic parameters (Table 1). This imply that simple recommendations from dietitians and fitness trainers were found to induce behavior changes, at least for 4 months. Therefore, in order to identify the metabolic parameters that were really influenced by the intervention in the LI group vs the SC group we have tested the significances of these delta of variations between the 2 groups and identified the metabolic parameters that were only influenced by the LI protocol (Table 1, significant delta). The results indicated that 4 months of intervention combining dietary modifications (low calories intake and introduction of high-fiber foods) and structured physical activities, induced a significant decrease in body weight, BMI and waist circumference. More importantly, the LI protocol improved insulin sensitivity, reduced fasting insulinemia, improve the level of the obesity-related adiponectin, decreased the level of the pro-inflammatory marker TNF-alpha and Il-6, and positively modulated the levels of exercise-induced myokines, irisin and BDNF. The LI protocol also decreased the anorexigenic gut peptide PYY (Table 1).

**Table 1:** Validation of the lifeStyle intervention protocol. Anthropometrics and metabolic parameters of the subjects involved in the Standard Care (SC) or LifeStyle Intervention protocol (LI) determined at 4 months and at 8 months post-intervention.

	Standard Care (SC)			LifeStyle Intervention (LI)			SIgnificant delta (LI-SC)	
	Basal	4 Mo	12 Mo	Basal	4 Mo	12 Mo	4 Mo	12 Mo
Weight (Kg)	71.67 ± 8.61	70.28 ± 8.75	70.35 ± 8.38	72.07 ± 8.82	66.17 ± 7.54 **	68.35 ± 9.32 **	**0.0004**	**0.005**
BMI (Kg/m²)	27.30 ± 2.88	26.67 ± 2.84 **	26.79 ± 2.70 *	27.9 ± 2.99	26.53 ± 2.83 **	26.52 ± 3.02 **	**0.0075**	**0.0076**
Waist circumference (cm)	93.13 ± 7.94	90.74 ± 8.73 **	90.74 ± 7.80 **	92 ± 9.2	87.28 ± 8.34 **	86.79 ± 8.69 **	0.0505	**0.0109**
hip-to-waist ratio	4.69 ± 1.16	4.40 ± 0.85	4.28 ± 1.08 *	4.79 ± 1.04	4.59 ± 0.87	4.32 ± 0.96 *	0.7344	0.7419
HOMA-IR	3.64 ± 1.67	3.55 ± 1.46	3.12 ± 1.40	2.61 ± 1.16	1.83 ± 0.83 **	1.95 ± 0.89 **	**0.0034**	0.2787
Fasting Insulinemia (mg/dl)	13.91 ± 5.99	14.03 ± 5.57	12.42 ± 5.51	10.37 ± 4.40	7.78 ± 3.49 **	8.29 ± 3.57 *	**0.0037**	0.3784
Fasting Glycaemia (mg/dl)	104.93 ± 9.41	101.37 ± 10.29	101.03 ± 8.81	101.14 ± 7.77	94.95 ± 6.59 **	94.55 ± 6.64 **	0.2209	0.1330
Hba1c (%)	6.07 ± 0.53	6.02 ± 0.48	6.03 ± 0.48	5.80 ± 0.47	5.65 ± 0.34	5.69 ± 0.27	0.2927	0.5117
Serum Cholesterol (mg/dl)	180.87 ± 33.81	175.63 ± 32.29	169.87 ± 33.58	189.5 ± 31.19	174.95 ± 30.57	179.18 ± 32.44	0.2611	0.9256
Serum Triglycerides (mg/dl)	135.87 ± 50.97	128.2 ± 37.20	125.57 ± 42.99	156.04 ± 125.39	130.73 ± 81.09	133.68 ± 105.29	0.371	0.4326
HDL cholesterol (mg/dl)	39.7 ± 7	40.43 ± 5.81	40.8 ± 7.59	40.55 ± 6.69	38.82 ± 6.1	42.59 ± 7.83	0.065	0.5356
LDL cholesterol (mg/dl)	113.97 ± 31.71	109.53 ± 27.43	103.93 ± 31.13	117.74 ± 31.59	110.05 ± 25.67	109.87 ± 30.19	0.6752	0.8023
VLDL cholesterol (mg/dl)	27.2 ± 10.25	25.67 ± 7.42	25.13 ± 8.62	31.21 ± 25.08	26.08 ± 16.21	26.72 ± 21.12	0.3435	0.4157
Bodyfat (%)	30.43 ± 7.60	29.56 ± 7.84	31.10 ± 7.83	30.08 ± 7.09	206.85 ± 57.93	30.95 ± 8.38	0.5297	0.9077
SBP (mmHg)	122.6 ± 15.28	117.6 ± 12.27	121.07 ± 14.69	121.72 ± 13.57	115.82 ± 15.65 **	118.14 ± 12.42	0.605	0.5019
DBP (mmHg)	72.83 ± 9.45	71.13 ± 7.10	70.5 ± 9.48	72.64 ± 9.08	68.5 ± 8.86	67.91 ± 7.89	0.2444	0.3745
Irisin (ng/ml)	39.52 ± 14.56	45.09 ± 18.72	51.22 ± 14.31	41.22 ± 16.81	61.42 ± 16.91 **	84.41 ± 30.02 **	**0.0316**	**0.0020**
BDNF (ng/ml)	813.49 ± 372.90	748.87 ± 342.66	919.66 ± 453.58	884.99 ± 298.31	1094.56 ± 265.75	1204.22 ± 435.44 **	**0.0294**	0.1391
Adiponectin (ng/ml)	237.57 ± 116.64	280.67 ± 147.31	244.5 ± 110.62	242.71 ± 105.29	359.63 ± 80.09 **	346.44 ± 113.69**	**0.0010**	**0.0018**
IL6 (ng/ml)	299.77 ± 61.52	272.43 ± 68.64	283.23 ± 82.39	306 ± 69.4	161.5 ± 42.56 **	207.95 ± 58.62 **	**0.0001**	**0.0001**
PYY (ng/ml)	13.28 ± 6.54	12.69 ± 6.44	12.39 ± 6.94	14.88 ± 5.99	10.34 ± 5.68 **	10.84 ± 4.23 *	**0.0166**	0.3022
TNF_alpha (ng/ml)v	32.29 ± 13.98	28.69 ± 11.09	28.82 ± 7.68	29.51 ± 9.4	18.03 ± 5.53 **	23.26 ± 9.97 **	**0.009**	0.1737
MCP (ng/ml)	547.39 ± 183.02	474.8 ± 186.79.	523.17 ± 191.76	539.91 ± 189.69	417 ± 145.89 **	402.82 ± 174.83 **	0.2788	**0.0458**
Leptin (ng/ml)	1085.5 ± 205.20	1039.2 ± 185.32	1063.97 ± 236.53	1041.09 ± 210.62	887.73 ± 175.71 *	896.45 ± 201.27 *	0.0552	0.09
Ghrelin (ng/ml)	204.23 ± 62.56	219.43 ± 60.99	202.03 ± 71.18	206.85 ± 57.93	241.33 ± 97.29 **	229.31 ± 97.55	0.3577	0.3347

Mo = Months. Data are adjusted to gender and age. Significant values calculated in each group independently are in bold (p<0.05 **;

p<0.01 ***). Significant variations (LI vs SC) = metabolic parameters which are affected by the LifeStyle Intervention protocol only (significant values are in bold). SBP = systolic blood pressure, DBP = Diastolic blood pressure.

### Lifestyle intervention modifies serum c-miRNA concentrations

**Fig 2:**
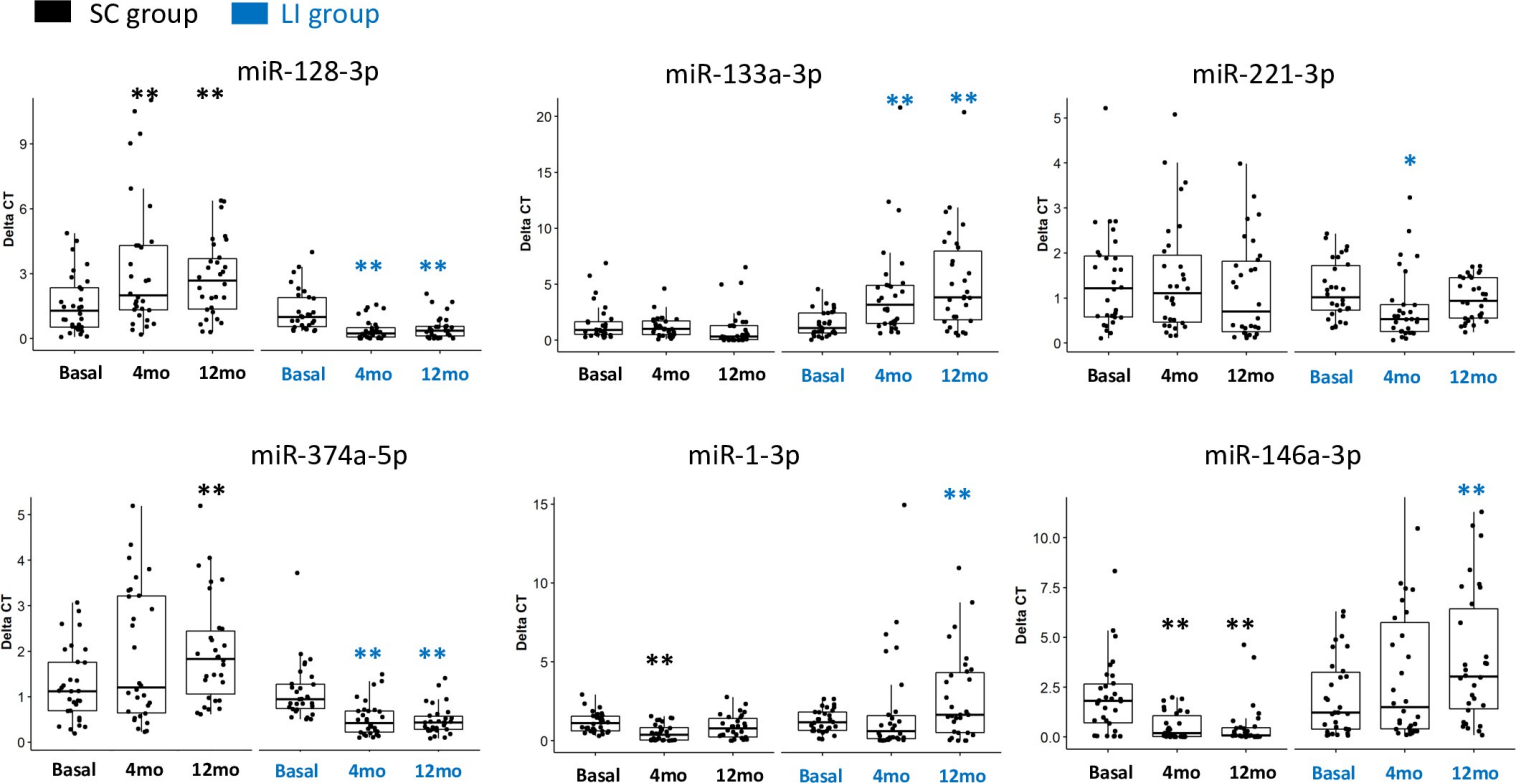
Expression of serum c-miRNAs significantly modulated by the 4 months LI protocol. Data are expressed as delta of Ct values inversely correlation with their blood concentrations. Gender and age were taken into account for the analyses.

**Fig 3:**
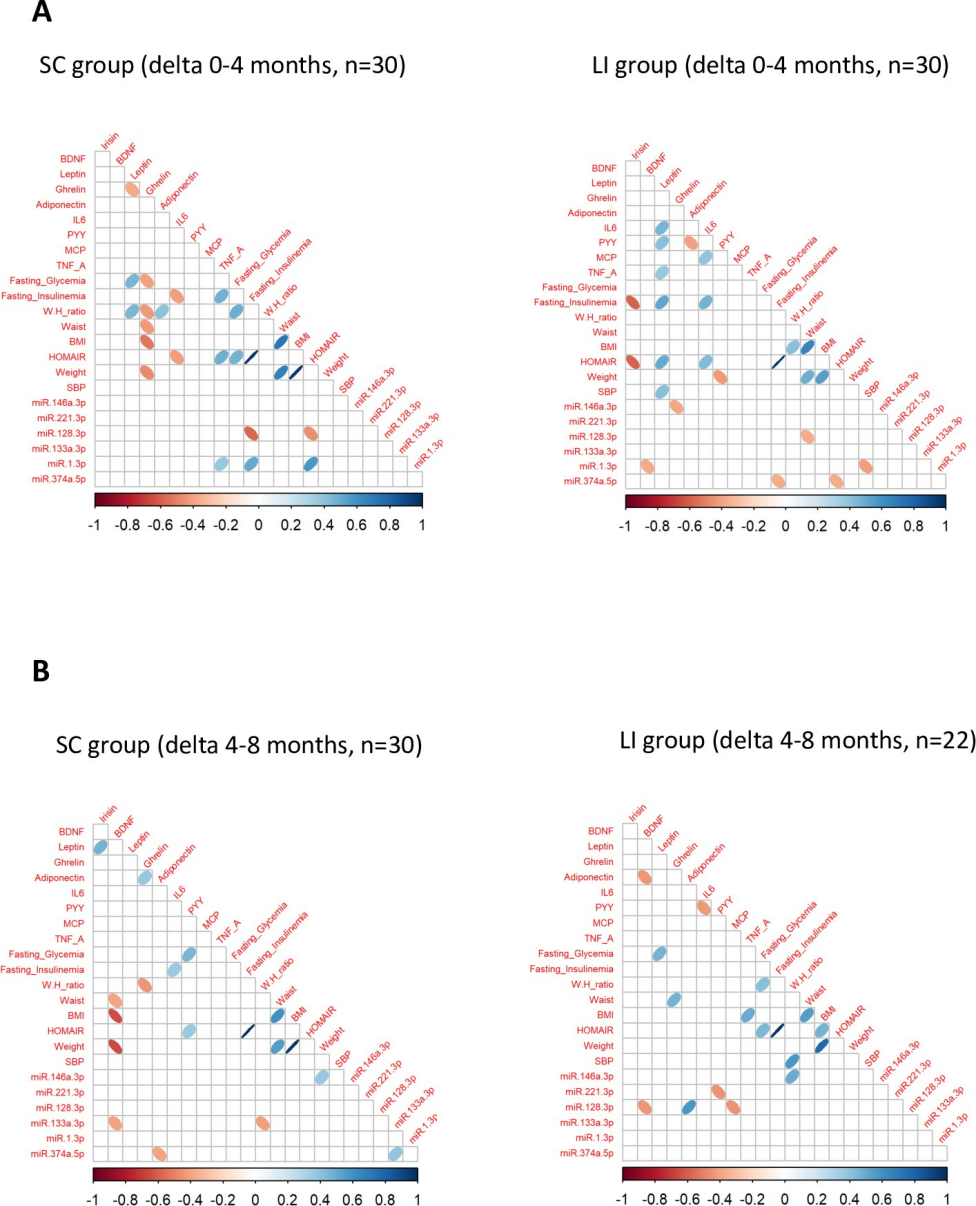
Spearman correlations between variations of c-miRNA levels and variations of metabolic parameters and cytokine concentrations. As after months, 8 patients from the LI group received metformin, they were removed from the analysis.

We first identified the c-miRNAs with significant variations within each group separately. Four months of LI intervention was associated with increase serum concentrations of miR-128-3p, miR-374a-5p and miR-221-3p, and a decrease of miR-133a-3p (Fig 2 and Table 2). MiR-128-3p, miR-374a-5p, and miR-1-3p and miR-146a-3p were also modulated in the SC group and interestingly, miR-128-3p, and miR-146a-3p were inversely regulated vs the LI group (Fig 2 and Table 2). Correlations between miRNA changes and metabolic parameters changes between basal and 4 months showed that miR-128-3p and miR-1-3p were correlated with the level of TNF-alpha, glycemia and insulin sensitivity (Fig 3A) suggesting that these 2 c-miRNAs could be early markers for metabolic deteriorations of SC group of patients. Indeed, we have previously identified miR-128-3p as specifically differentially expressed in blood of subjects from the ’Asian Indian Phenotype’ with impaired glucose tolerance, compared to control subjects [5].

**Table 2:** Variations of circulating miRNAs (ratios of Ct values) in serum from all subjects involved in the Standard Care (SC) or LifeStyle Intervention protocol (LI).

	Standard Care			LifeStyle Intervention			SIgnificant variations (LI-SC)	
	Basal	4 Mo	12 Mo	Basal	4 Mo	12 Mo	4 Mo	12 Mo
miR-128-3p	1.59 ± 1.36	3.36 ± 3.14 **	2.78 ± 1.76 **	1.29 ± 0.98	0.42 ± 0.48 **	0.52 ± 0.55 **	**0.0001**	**0.0001**
miR-133a-3p	1.53 ± 1.64	1.20 ± 0.94	1.07 ± 1.66	1.47 ± 1.14	4.29 ± 4.29 **	5.21 ± 4.49 **	**0.0004**	**0.0001**
miR-146a-3p	1.99 ± 1.98	0.54 ± 0.66 **	0.55 ± 1.09 **	2.02 ± 1.94	3.14 ± 3.44	3.91 ± 3.33 **	**0.0009**	**0.0001**
miR-423-5p	1.69 ± 1.74	1.09 ± 0.94	0.96 ± 0.68	1.50 ± 1.25	1.98 ± 0.64 *	1.69 ± 0.88	0.0076	**0.0266**
miR-374a-5p	1.26 ± 0.81	1.90 ± 1.47	1.99 ± 1.16 **	1.12 ± 0.64	0.52 ± 0.38 **	0.48 ± 0.32 **	**0.0001**	**0.0001**
miR-1-3p	1.16 ± 0.63	0.51 ± 0.52 *	0.86 ± 0.75	1.26 ± 0.73	1.93 ± 3.26	2.78 ± 2.80 *	**0.0555**	**0.0029**
let7d-3p	1.55 ± 1.37	0.95 ± 1.01	1.20 ± 1.30	1.25 ± 0.83	1.47 ± 0.97	1.78 ± 1.37	0.0205	0.2308
miR-126-3p	1.33 ± 1.02	1.11 ± 0.41	1.68 ± 1.52	1.29 ± 0.81	1.68 ± 0.79	2.07 ± 1.53	0.0811	0.1139
miR-21-5p	1.37 ± 0.93	1.92 ± 1.19	1.87 ± 2.35	1.47 ± 1.09	0.97 ± 0.59	1 ± 0.55	0.0140	0.9947
miR-222-3p	2.02 ± 2.11	1.92 ± 1.89	1.91 ± 1.66	1.58 ± 1.47	0.75 ± 0.56	1.04 ± 0.71	0.1534	0.4090
miR-221-3p	1.39 ± 1.09	1.44 ± 1.25	1.18 ± 1.58	1.17 ± 0.64	0.79 ± 0.77 *	0.97 ± 0.47	**0.0471**	0.4702
miR-210-3p	2.27 ± 2.29	2.92 ± 2.45	3.48 ± 4.05	1.86 ± 2.36	1.08 ± 1.12	1.87 ± 1.62	0.0055	0.6526
miR-130b-3p	1.15 ± 0.59	0.98 ± 0.82	1.44 ± 1.08	1.81 ± 2.97	0.84 ± 0.56	1.28 ± 0.77	0.8281	0.7087
miR-29b	1.50 ± 1.44	1.27 ± 1.05	1.58 ± 1.50	1.32 ± 1	0.95 ± 0.52	0.94 ± 0.43	0.6969	0.8244
miR-29c	1.32 ± 0.87	1.54 ± 1	1.18 ± 1.07	1.76 ± 1.66	2.15 ± 2.82	1.67 ± 1.67	0.4723	0.3892

Mo = Months. Data were adjusted for gender and age. Ct values are inversely correlated with seric miRNAs concentrations. Significant values calculated in each group independently are in bold (p<0.05 *;

p<0.01 ** (4 months vs Basal or 12months vs Basal)). Significant variations (delta LI vs delta SC) = list of miRNAs affected by the LifeStyle Intervention protocol only.

The significant regulation of miR-423-5p at 4 months was lost when the 8 subjects treated with metformin were removed from the LI group before analysis. The significant variation (delta LI vs delta SC) were lost for miR-21-5p when the 8 subjects treated with metformin were removed from the LI group before analysis.

Then, as for the metabolic data, we then used statistical tests to identify c-miRNA variations significantly associated with the LI protocol. Results indicated that 4 months of LI intervention was associated with increase serum concentrations of miR-128-3p, miR-374a-5p and miR-221-3p, and a decrease of miR-133a-3p (Fig 2 and Table 2, significant delta). Although indicated as differentially modulated between the LI and SC groups, let-7-3p and miR-210-3p were not significantly modulated inside each group, likely because of their high variability among the individuals. They were not considered in Fig 2. As shown on Fig 3B, miR-374a-5p was negatively correlated with fasting insulinemia and HOMA-IR, 2 metabolic parameters ameliorated during the LI protocol (Table 1). MiR-128-3p variations were negatively correlated with the decrease of waist circumference in the LI group, miR-1-3p variations were negatively correlated with BDNF and systolic blood pressure, and miR-146a-3p was negatively correlated with the variations of ghrelin (Fig 2). Thus, the 4 months of LI modulated c-miRNAs and these variations were correlated with metabolic parameters ameliorated by LI.

### Height months post-LI, c-miRNAs were still positively modified in serum of pre-diabetic subjects although the benefice of the LI protocol was lost

As there is now emerging data demonstrating that intensive lifestyle modification, even for a limited period of time, can have long-term benefits, as far as the risk of type 2 diabetes is concerned [16, 21], blood samples were collected 8 months post-LI in order to determine the long term consequences of the protocol on both metabolic parameters and c-miRNA levels. During the 8 months post-LI, clinical staff contact with all participants was minimal, but health educators were available to answer questions and to hold occasional exercise refresher courses (Fig 1). After one year, the metabolic parameters and the concentrations of c-miRNAs were compared versus their levels at the beginning of the protocol. Many of the metabolic parameters ameliorated during the 4-month intervention declined when the protocol stopped and returned to basal state 8-month post LI, except for BMI, waist circumference and hip-to-waist ratio (Table 1) suggesting that the volunteers did not follow the LI protocol on their own. However, the levels of 3 cytokines (i.e.; Irisin, IL-6 and adiponectin) continued to be significantly and beneficially modified one year post-LI (Table 1, significant delta) [22]. Interestingly, the level of the inflammatory marker MCP-1 continue to decrease and was significantly reduced 8 months post-LI vs basal state. More importantly, serum concentrations of miR-128-3p, miR-133a-5p, miR-374a-5p and miR-1-3p did not return to basal state 8-month post LI, and the level of miR-146a continued to decrease and reached the level of significance (Table 2 and Fig 2). In addition, between 4 and 8 months follow-up, serum variations of miR-128-3p was negatively correlated with variations of BDNF and MCP and positively with variation of adiponectin, and miR-146a-3p variations were negatively correlated with hip-to-waist ratio (Fig 3B).

## Discussion

It was previously demonstrated that renormalization of glycemia with insulin-sensitizer or gut surgery was associated with the renormalization of specific c-miRNA concentrations toward their levels in normoglycemia state suggesting that the level of c-miRNAs might be associated with alterations of specific metabolic parameters linked to glucose homeostasis and insulin sensitivity [3, 15]. Based on these data it has been admitted that c-miRNAs could be used as biomarkers of metabolic and insulin-sensitivity improvement. In this study, we have challenged this hypothesis and analysed the variations of c-miRNAs from obese pre-diabetic individuals at risk of developing diabetes, enrolled in a structured lifestyle (LI) protocol combining weight loss, diet modification and moderate level of physical activity [16, 20]. As control, a similar group of obese pre-diabetic individuals was enrolled to determine the evolution of the c-miRNAs without intervention, during the same period of time (SC). As expected, our data showed that 4 months post-intervention, all subjects had significant metabolic improvement compared to the control group confirming the positive impact of the LI protocol on insulin-sensitivity [20]. However, most of these metabolic parameters were not different from the basal values one year post-intervention suggesting that the patients did not continue the protocol on their own. Therefore, in order to determine whether variations of c-miRNAs could be used to follow this metabolic deterioration, we have considered 2 periods of time to analyse the variations of c-miRNAs, i.e.; the first was to study the dynamic response to the intervention (4 months), and the second represented the 8 months post-intervention. After 4 months, the levels of 4 miRNAs (i.e.; miR-128-3p, miR-374a-5p, miR-221-3p, and miR-133a-3p) were changed only in the LI group. Among them, miR-128-3p had previously been identified altered in the blood from pre-diabetic vs healthy subjects, and miR-374a-5p was altered in the blood of diabetic vs pre-diabetic subjects [5]. In this study, although not significant, their variations in blood from the standard care group were inversely correlated to those of LI group, demonstrating that the protocol could prevent their ’natural’ deterioration in blood. As our previous results on the same D-CLIP cohort showed that this intervention has reduced the 1-year incidence of diabetes by half compared with the control group [23], these data indicated thus that c-miRNA variations, during the 4 month of intervention, directly reflected the amelioration of whole-body glucose homeostasis and insulin-sensitivity of the pre-diabetic patients. More particularly, the blood alteration of c-miR-128 level, which is only identified in this specific Indian population (11), was modulated by the intervention and we suggest that it could be used, in addition to metabolic/cytokine parameters, as an early marker of metabolic improvement.

Therefore, based on these results, we expected that the metabolic deterioration of the patients during the post-intervention period, would impact blood variations of these 4 c-miRNAs. Very surprisingly, 8 months post-LI, the positive effects of the intervention on the c-miRNA levels observed at 4 months continued to be detected and were even more pronounced for miR-128-3p, miR-133a-3p, miR-146a-3p, miR-374a-5p and miR-1-3p, as suggested by significant lower p-values at 8 vs 4 months. In addition, the levels of 3 cytokines remained significantly different from the basal state (i.e.; the exercise-induced Irisin, IL-6 and adiponectin negatively correlated with adipose tissue expansion). These unexpected results indicated clearly that conversely to what is generally admitted, c-miRNA variations are not directly correlated with variations of metabolic/anthropometric parameters and clearly, they cannot be considered as biomarkers of glucose homeostasis and insulin sensitivity. In this study, the significant correlations obtained at basal state between c-miRNAs and some metabolic data which were significant after the intervention, were lost after 4 months, and other correlations with different metabolic parameters appeared post-intervention.

This important result might explain the incoherent data between the previously published studies designed to identify specific c-miRNA associated to the severity of insulin-resistance or with different states of diabetes development. The subset of miRNAs claimed as biomarkers of specific metabolic alteration or related to glucose homeostasis is never the same across the studies [3, 4]. One possible explanation would be that the level of physical activity and the quality of the diet have rarely been taken into account in these comparative studies. In addition, these studies compared only two situations: i.e.; insulin-resistant vs NGT subjects; or after/before the use of insulin-sensitizers, or physical activity, or obesity surgery, or specific diet, vs untreated controls without looking at how these c-miRNAs vary over the time after the interventions. Clearly, our data indicate that even if c-miRNAs are modulated by the lifestyle intervention, the origin of their blood variations is difficult to explain if we cannot identify the origin of their presence in blood. It was suggested that the great majority of c-miRNAs are packed inside extracellular vesicles (EVs) [24] and that these vesicles are mainly released from blood cells [25]. Indeed, the miRNA signatures of PBMC and blood EVs are very similar [25], and miRNA levels from PBMC are strongly correlated with the metabolic status of the diabetic patients [26–29]. Therefore, if we consider that the majority of c-miRNAs are originated from PBMCs, c-miRNAs correlations with metabolic parameters would be indirect, but would directly reflect the level of systemic inflammation associated with the obesity-induced pre-diabetic state. In line with this suggestion, beside the decrease of waist-circumference associated with visceral adiposity and the increased level of adiponectin [30], the other important marker which was still positively reduced during 8 months post-intervention was the level of Monocyte Chemoattractant Protein-1 (MCP-1/CCL2), one of the key chemokines that regulate migration and infiltration of monocytes/macrophages in inflamed tissues [31]. In addition, the levels of 2 cytokines also markers of inflammation such as irisin, and Il-6 [32, 33] were also significantly different between 8 months post-LI and basal state, confirming that the LI protocol had a strong impact on the level of inflammation even 8 months post intervention. A recent meta-analysis, published after the completion of our manuscript, to identify biomarkers of type 2 diabetes, indicated that miR-155 was consistently found in all studies [4]. Interestingly, miR-155 has been found to participate in the deleterious cross-talk between immune cells and pancreas or adipose tissue [24, 34] and its circulating level is correlated with waist-circumference level [35] which is the only anthropometric data significantly different from basal state after 8 months post-LI in this study. As the beneficial effect of the lifestyle intervention, and more particularly of the exercise training, have long-time effects on inflammation [36], it would explain why in our study, c-miRNAs levels are disconnected from the metabolic parameter variations and insulin sensitivity.

In conclusion, this study confirmed that c-miRNA levels are altered in obese pre-diabetic individuals with the alterations of glucose homeostasis and insulin-sensitivity, and that lifestyle modifications can modulate c-miRNA concentrations and restore their levels, as in healthy subjects. However, we observed a disconnection between c-miRNA variations and metabolic/anthropometric parameters variations when the intervention was stopped suggesting that these correlations are not direct. We suggest that c-miRNA levels are in fact directly correlate with the level of systemic inflammation. More generally speaking, we think that this important result explains the high variability between the previous studies designed to identify specific c-miRNAs associated with the severity of diabetes. The results of all these studies should take into account the level of inflammation of the individuals (9). Finally, it could also explain why, whatever the pathology considered (i.e.; cancers, diabetes, neurodegenerative disorders, inflammatory diseases) the same subset of miRNAs is always found altered in the blood of patients vs control subjects, as these pathologies are all associated with the development of inflammation.

## Supporting information

S1 Table Clinical and biochemical characteristics of the subjects involved in the study (n = 60). (PDF)

S2 Table Metabolic and clinical data of the women involved in the study at basal state (n = 27). (PDF)

S3 Table Metabolic and clinical data of the men involved in the study at basal state (n = 33). (PDF)

S1 Fig Principal component analyses taking into account all variables (miRNA levels and anthropometric and metabolic parameters) for T0, T = 4 months, and T = 8 months. The subjects who received metformin after 4 months intervention (in red) are not different from the subjects without metformin (in green).
(PDF)
