# Correction: Blood-derived miRNA levels are not correlated with metabolic or anthropometric parameters in obese pre-diabetic subjects but with systemic inflammation

**DOI:** 10.1371/journal.pone.0272323

**Published:** 2022-07-26

**Authors:** Prabu Paramasivam, Emmanuelle Meugnier, Kuppan Gokulakrishnan, Harish Ranjani, Lisa R. Staimez, Mary Beth Weber, K. M. Venkat Narayan, Hubert Vidal, Nikhil Tandon, Dorairaj Prabhakaran, Ranjit Mohan Anjana, Viswanathan Mohan, Sophie Rome, Muthuswamy Balasubramanyam

The fourth and eleventh authors’ names are spelled incorrectly. The fourth author’s correct name is: Harish Ranjani. The eleventh author’s correct name is: Ranjit Mohan Anjana. The correct citation is: Paramasivam P, Meugnier E, Gokulakrishnan K, Ranjani H, Staimez LR, Weber MB, et al. (2022) Blood-derived miRNA levels are not correlated with metabolic or anthropometric parameters in obese pre-diabetic subjects but with systemic inflammation. PLoS ONE 17(2): e0263479. https://doi.org/10.1371/journal.pone.0263479

The affiliations for the seventh and ninth authors are incorrect. K. M. Venkat Narayan is not affiliated with #6 but with #5: Emory University, Atlanta, GA, United States of America. Nikhil Tandon is not affiliated with #5 but with #6: Department of Endocrinology, All India Institute of Medical Sciences, New Delhi, India.
