# Supplementary material for: Blood-derived miRNA levels are not correlated with metabolic or anthropometric parameters in obese pre-diabetic subjects but with systemic inflammation
Source: PLoS One. 2022 Feb 4;17(2):e0263479. doi: 10.1371/journal.pone.0263479 (PMC8815902; doi:10.1371/journal.pone.0263479)
Supplement: S1 Table — (PDF) [file pone.0263479.s001.pdf]

Table S1. Clinical and biochemical characteristics of the subjects involved in the study (n=60) at basal state (*= *p* <0.05, standard group *vs* intervention group)

Parameters	Mean ± SEM Standard Care	Mean ± SEM Lifestyle intervention	Student't test *p* values	BH corrected p values
Females/males	(53%/47%)	(37%/63%)	0.19	0.93275
Age (years)	43.7 ± 1.57	48.73 ± 1.46	0.023*	0.598
BMI (Kg/m2)	27.29 ± 0.52	28.11 ± 0.75	0.38	0.93275
Body Weight (Kg)	71.67 ± 1.57	73.48 ± 1.92	0.47	0.93275
Waist circumference (cm)	93.13 ± 1.44	94.42 ± 1.91	0.592	0.93275
ratios (cm)	4.68 ± 0.21	4.70 ± 0.20	0.964	0.971
Fasting Insulin (mIU/ml)	13.91 ± 1.09	11.39 ± 1.28	0.141	0.93275
Fasting plasma glucose (mg/dl)	104.93 ± 1.71	103.3 ± 1.48	0.474	0.93275
HOMA-IR	3.64 ± 0.30	2.94 ± 0.35	0.135	0.93275
Fat Mass (%)	30.42 ± 1.38	29.67 ± 1.33	0.696	0.93275
Systolic blood pressure (mmHg)	122.6 ± 2.79	122.47 ± 2.33	0.971	0.971
Diatolic blood pressure (mmHg)	72.83 ± 1.72	73,93 ± 1.60	0.641	0.93275
HbA1c (%)	6.06 ± 0.09	5.93 ± 0.09	0.301	0.93275
Serum Cholesterol (mg/dl)	180.86 ± 6.17	186.20 ± 6.03	0.539	0.93275
Serum Triglycerides (mg/dl)	135.86 ± 9.30	150.04 ± 19.83	0.52	0.93275
HDL cholesterol (mg/dl)	39.7 ± 1.27	40.57 ± 1.16	0.618	0.93275
LDL cholesterol (mg/dl)	113.96 ± 5.78	115.65 ± 5.89	0.84	0.93275
VLDL cholesterol (mg/dl)	27.2 ± 1.87	29.99 ± 3.97	0.529	0.93275
Leptin	1085.5 ± 37.46	1045.5 ± 40,63	0.468	0.93275
Ghrelin	204.22 ± 11.42	197,51 ± 10,48	0.666	0.93275
Adiponectin	237.56 ± 21.29	304.13 ± 13.88	0.809	0.93275
Il-6	299.76 ± 11.23	299.76 ± 11.23	0.808	0.93275
PYY	13.28 ± 1.19	14,82 ± 1.29	0.388	0.93275
MCP	547.39 ± 33.41	556.17 ± 37.17	0.861	0.93275
TNF-ALPHA	32.29 ± 2.55	31.62 ± 2.11	0.841	0.93275
