# Supplementary material for: Blood-derived miRNA levels are not correlated with metabolic or anthropometric parameters in obese pre-diabetic subjects but with systemic inflammation
Source: PLoS One. 2022 Feb 4;17(2):e0263479. doi: 10.1371/journal.pone.0263479 (PMC8815902; doi:10.1371/journal.pone.0263479)
Supplement: S2 Table — (PDF) [file pone.0263479.s002.pdf]

Table S2: Metabolic and clinical data of the women involved in the study at basal state (n=27) (*= p<0.05, standard care group vs intervention group)

Parameters	Mean ± SEM Standard Care	Mean ± SEM Lifestyle intervention	Student t test *p* values
Age (years)	46 ± 2.301	44 + 2.941	0.146
BMI (Kg/m^2)	28 + 0.963	30 + 1.379	0.214
Body Weight (Kg)	67 + 2.266	69 + 3.116	0.463
Waist circumference (cm)	88 + 1.915	89 + 4.040	0.775
ratios (cm)	4.418 + 0.318	4.582 + 0.332	0.704
Fasting Insulin (mlU/ml)	15.187 + 2.191	11.063 + 1.407	0.085
Fasting plasma glucose (mg/dL)	104.562 + 2.638	101.818 + 2.066	0.371
HOMA-IR	3.989 + 0.620	2.806 + 0.385	0.079
Fat Mass (%)	36.331 + 1.411	37.054 + 1.097	0.656
Systolic blood pressure (mmHg)	124.812 + 5.704	118 + 4.559	0.310
Diatolic blood pressure (mmHg)	71.75 + 3.228	70.182 + 2.756	0.687
HbA1c (%)	6.031 + 0.160	5.820 + 0.186	0.362
Serum Cholesterol (mg/dL)	180.562 + 9.491	193.636 + 10.383	0.327
Serum Triglycerides (mg/dL)	136.875 + 16.893	128 + 17.873	0.700
LDL (mg/dl)	111 + 8.775	124.820 + 9.525	0.262
HDL cholesterol (mg/dL)	42.125 + 2.162	43.182 + 1.600	0.664
LDL cholesterol (mg/dL)	111 + 8.775	124.820 + 9.525	0.262
VLDL cholesterol (mg/dL)	27.437 + 3.382	25.636 + 3.543	0.694
Leptin (ng/mL)	1111.812 + 77.113	1044.910 + 66.688	0.476
Ghrelin (ng/mL)	185.616 + 22.910	196.830 + 17.493	0.668
Adiponectin (ng/mL)	243.122 + 39.273	223.818 + 34.247	0.687
Il-6 (ng/mL)	296.375 + 18.705	322 + 20.327	0.327
PYY (ng/mL)	14.029 + 2.520	15.794 + 2.231	0.569
MCP (ng/mL)	587.737 + 58.490	617.910 + 62.896	0.708
IRISIN (ng/mL)	40.37 + 4.14	46.98 + 6.68	0.412
BDNF (ng/mL)	838.54 + 62.15	902.01 + 90.47	0.453
TNF-ALPHA (ng/mL)	32.181 + 4.363	34.582 + 3.146	0.621
