# Supplementary material for: Blood-derived miRNA levels are not correlated with metabolic or anthropometric parameters in obese pre-diabetic subjects but with systemic inflammation
Source: PLoS One. 2022 Feb 4;17(2):e0263479. doi: 10.1371/journal.pone.0263479 (PMC8815902; doi:10.1371/journal.pone.0263479)
Supplement: S3 Table — (PDF) [file pone.0263479.s003.pdf]

Table S3: Metabolic and clinical data of the men involved in the study at basal state (n=33)
(*= p<0.05, standard car group vs intervention group)

Parameters	Mean + SEM Standard Care	Mean + SEM Lifestyle intervention	Student't test *p* values
Age (years)	40.928 + 2.440	51.368 + 1.254	0.0011*
BMI (Kg/m^2)	26.485 + 0.618	26.974 + 0.794	0.630
Body Weight (Kg)	76.771 + 1.185	75.521 + 2.365	0.680
Waist circumference (cm)	98 + 1.407	97 + 1.669	0.493
ratios (cm)	4.993 + 0.331	4.768 + 0.260	0.596
Fasting Insulin (mlU/ml)	12.457 + 1.037	11.584 + 1.883	0.688
Fasting plasma glucose (mg/dL)	105.357 + 2.790	104.158 + 2.019	0.731
HOMA-IR	3.247 + 0.272	3.015 + 0.510	0.692
Fat Mass (%)	23.678 + 0.915	25.4 + 1.149	0.250
Systolic blood pressure (mmHg)	120.071 + 2.607	125.053 + 2.469	0.175
Diatolic blood pressure (mmHg)	74.071 + 2.134	76.105 + 1.822	0.475
HbA1c (%)	6.107 + 0.146	5.989 + 0.100	0.513
Serum Cholesterol (mg/dL)	181.214 + 10.022	181.895 + 7.412	0.957
Serum Triglycerides (mg/dL)	134.714 + 12.452	162.842 + 29.544	0.389
LDL (mg/dl)	117.357 + 9.421	114.941 + 6.352	0.836
HDL cholesterol (mg/dL)	36.928 + 1.574	39.053 + 1.503	0.337
LDL cholesterol (mg/dL)	117.357 + 9.421	114.941 + 6.351	0.836
VLDL cholesterol (mg/dL)	26.923 + 2.519	24.294 + 1.888	0.420
Leptin	1055.428 + 34.462	1045.316 + 52.627	0.873
Ghrelin	225.496 + 9.082	197.899 + 13.460	0.099
Adiponectin	231.222 + 27.633	234.337 + 25.154	0.934
Il-6	303.643 + 16.846	293.789 + 18.480	0.696
PYY	12.434 + 0.980	14.250 + 1.608	0.343
IRISIN	38.56 + 3.32	39.59 + 3.84	0.830
BDNF	798.85 + 110.823	902.01 + 90.467	0.453
MCP	501.286 + 43.910	520.421 + 45.253	0.763
TNF-ALPHA	32.414 + 3.726	29.910 + 2.770	0.594
