# Supplementary material for: Blood-derived miRNA levels are not correlated with metabolic or anthropometric parameters in obese pre-diabetic subjects but with systemic inflammation
Source: PLoS One. 2022 Feb 4;17(2):e0263479. doi: 10.1371/journal.pone.0263479 (PMC8815902; doi:10.1371/journal.pone.0263479)
Supplement: S1 Fig — The subjects who received metformin after 4 months intervention (in red) are not different from the subjects without metformin (in green). (PDF) [file pone.0263479.s004.pdf]

Supplementary Figure S1

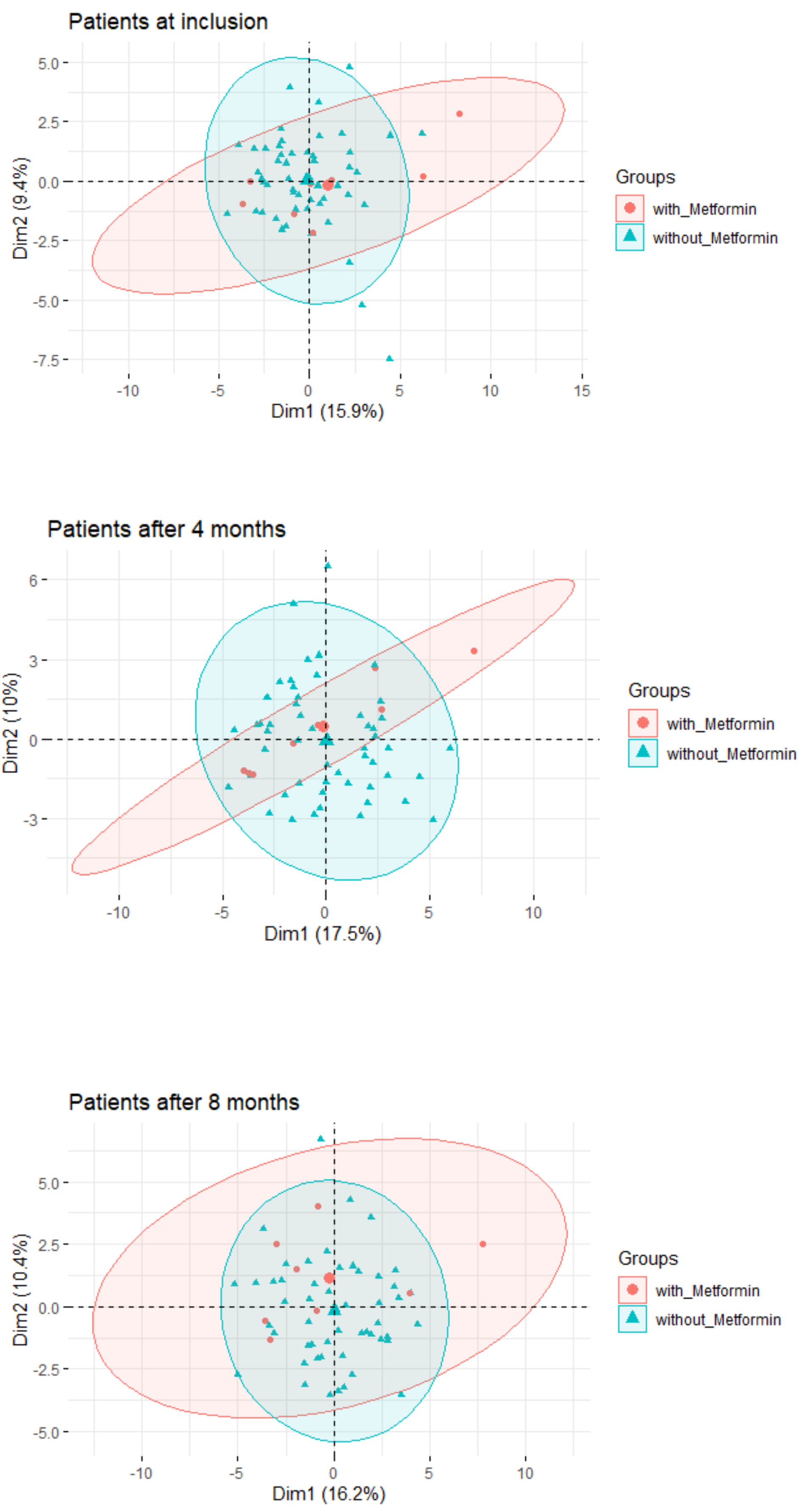

Legend: Principal Component Analyses taking into account all variables (miRNA levels and anthropometric and metabolic parameters) for T0, T= 4 months, and T= 8 months. The subjects who received metformin after 4 months intervention (in red) are not different from the subjects without metformin (in green).
